# Artificial intelligence in chronic kidney disease management: a scoping review

**DOI:** 10.7150/thno.108552

**Published:** 2025-03-21

**Authors:** Charumathi Sabanayagam, Riswana Banu, Cynthia Lim, Yih Chung Tham, Ching-Yu Cheng, Gavin Tan, Elif Ekinci, Bin Sheng, Gareth McKay, Jonathan E. Shaw, Kunihiro Matsushita, Navdeep Tangri, Jason Choo, Tien Y. Wong

**Affiliations:** 1 Singapore Eye Research Institute, Singapore National Eye Centre, Singapore.; 2 Ophthalmology and Visual Science Academic Clinical Program, Duke-NUS Medical School, Singapore.; 3 Saw Swee Hock School of Public Health, National University of Singapore, Singapore.; 4 Department of Renal Medicine, Singapore General Hospital, Singapore.; 5 Department of Ophthalmology, Yong Loo Lin School of Medicine, National University of Singapore, Singapore.; 6 Department of Medicine, Melbourne Medical School, The University of Melbourne, Australia and Department of Endocrinology, Austin Health, Melbourne, Australia.; 7 Department of Computer Science and Engineering, Shanghai Jiao Tong University, Shanghai, China.; 8 Centre for Public Health, Queen's University Belfast, Northern Ireland, United Kingdom.; 9 Baker Heart and Diabetes Institute, Melbourne, Victoria, Australia.; 10 Department of Epidemiology, Johns Hopkins Bloomberg School of Public Health, Baltimore, Maryland, USA.; 11 Department of Medicine and Community Health Sciences, University of Manitoba, Winnipeg, Manitoba, Canada.; 12 School of Clinical Medicine, Beijing Tsinghua Changgung Hospital, Tsinghua Medicine, Tsinghua University, Beijing, China.

**Keywords:** machine learning, deep learning, clustering, large language models, early detection, risk stratification, patient care

## Abstract

**Rationale:** Chronic kidney disease (CKD) is a major public health problem worldwide associated with cardiovascular disease, renal failure, and mortality. To effectively address this growing burden, innovative solutions to management are urgently required. We conducted a scoping review to identify key use cases in which artificial intelligence (AI) could be leveraged for improving management of CKD. Additionally, we examined the challenges faced by AI in CKD management, proposed potential solutions to overcome these barriers.

**Methods:** We reviewed 41 articles published between 2014-2024 which examined various AI techniques including machine learning (ML) and deep learning (DL), unsupervised clustering, digital twin, natural language processing (NLP) and large language models (LLMs) in CKD management. We focused on four areas: early detection, risk stratification and prediction, treatment recommendations and patient care and communication.

**Results:** We identified 41 articles published between 2014-2024 that assessed image-based DL models for early detection (n = 6), ML models for risk stratification and prediction (n = 14) and treatment recommendations (n = 4), and NLP and LLMs for patient care and communication (n = 17). Key challenges in integrating AI models into healthcare include technical issues such as data quality and access, model accuracy, and interpretability, alongside adoption barriers like workflow integration, user training, and regulatory approval.

**Conclusions:** There is tremendous potential of integrating AI into clinical care of CKD patients to enable early detection, prediction, and improved patient outcomes. Collaboration among healthcare providers, researchers, regulators, and industries is crucial to developing robust protocols that ensure compliance with legal standards, while minimizing risks and maintaining patient safety.

## Introduction

Chronic kidney disease (CKD) is a global health challenge associated with serious adverse consequences including cardiovascular disease (CVD), kidney failure, premature death, and poor quality of life. A recent meta-analysis reported the global prevalence of CKD to be 11%-13% affecting an estimated 844 million people worldwide, twice the estimated number of people with diabetes 1. With aging populations and increasing prevalence of diabetes and hypertension, the burden of CKD is projected to increase substantially. CKD was ranked as the 12^th^ leading cause of death accounting for 1.2 million deaths in 2017 with this number expected to reach 4 million and the 5^th^ leading cause of death by 2040 2, 3. Developed nations allocate over 2-3% of their annual healthcare budget to treat end-stage kidney disease (ESKD), despite it affecting only 0.02-0.03% of the total population. To effectively address this growing burden of CKD, innovative solutions are urgently required.

In the past decade, a series of new artificial intelligence (AI) technologies, including machine learning (ML) and deep learning (DL), natural language processing (NLP) and large language models (LLMs), have been developed, offering highly promising approaches to optimize CKD management. Previous reviews on AI in kidney disease have either addressed AI in nephrology as a broad topic 4, covering areas such as acute kidney injury (AKI) 5, CKD, renal cancer, dialysis, and transplantation management 6, 7, or focused on specific techniques, such as traditional ML models 8, 9 or NLP 10. There has been no comprehensive review of emerging AI technologies, such as DL, unsupervised learning, and LLMs in CKD management. In this review, we address these gaps by exploring a full range of AI techniques beyond traditional ML, including DL, unsupervised clustering, NLP, and LLMs (ChatGPT) in CKD management. Additionally, we examine technical and non-technical challenges to integrating AI into CKD management and propose potential solutions.

**Figure 1:**
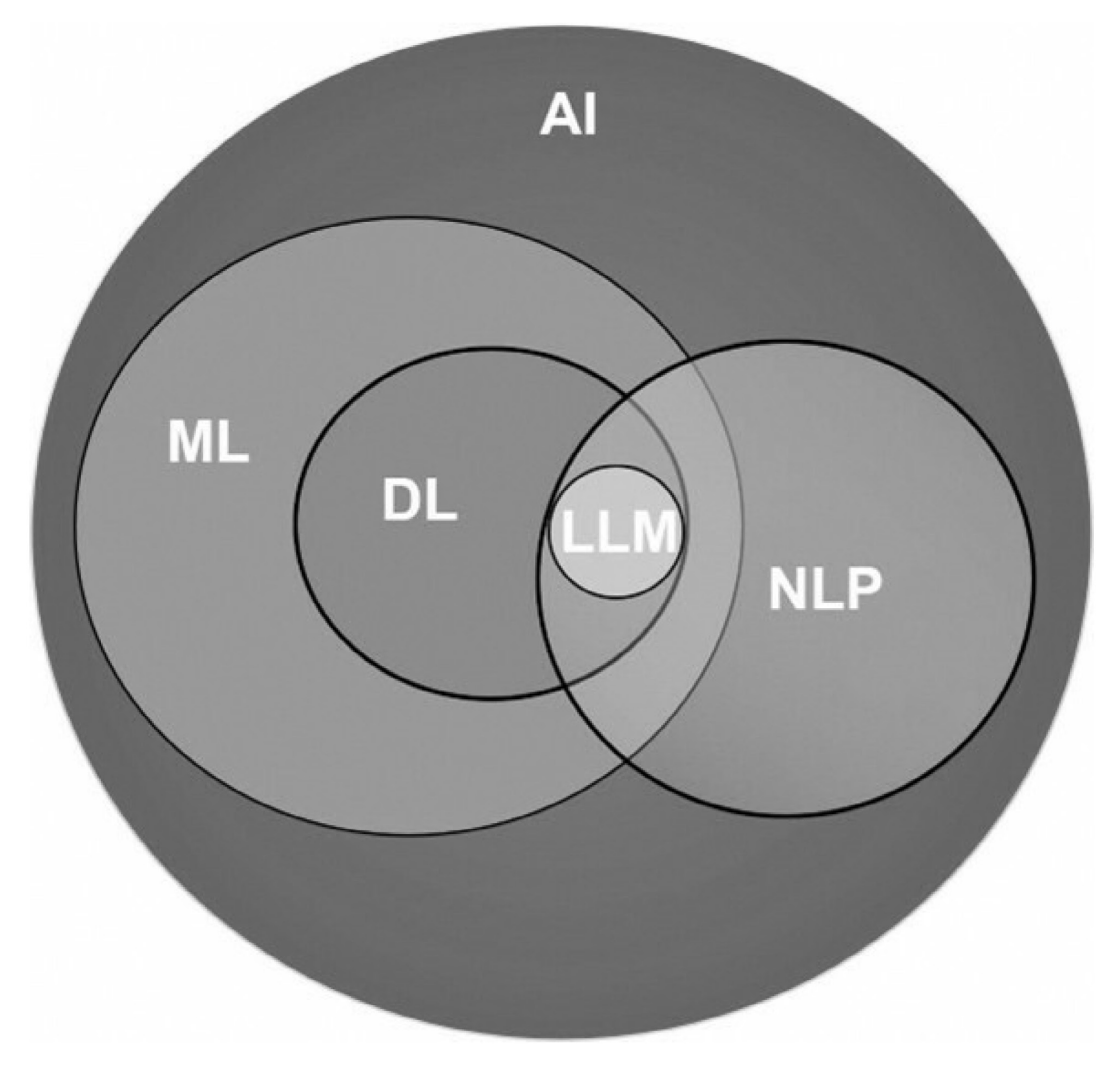
Artificial intelligence and its subfields.

**Figure 1** illustrates the expansive field of AI and its pivotal subfields that significantly contribute to advancing healthcare practices. AI is a computer science field focused on creating intelligent machines capable of mimicking human cognitive functions like learning, decision making and problem-solving but offers faster processing, potentially more objective decision-making, and the ability to analyze vast amounts of data that would be unmanageable for humans. ML, a subset of AI, focuses on enabling computers to learn from data without explicit programming, allowing them to improve performance through experience. DL, a subfield of ML, utilizes artificial neural networks (ANN) inspired by the structure of the human brain. These networks have multiple layers and are proficient in processing complex forms of data, such as images, text, or speech. NLP, is a subfield of AI that deals with the interaction between computers and human language allowing computers to understand, interpret, and generate human language. LLMs, a subfield of DL, excels in understanding, reasoning and generating human-like text.

ML, one of the cornerstones of AI, can be broadly classified into several types, each serving different purposes (**Table 1**). Supervised learning relies on labeled data to train models that can predict outcomes or classify information, while unsupervised learning identifies patterns in unlabeled data. Reinforcement learning uses trial-and-error methods to optimize decisions, particularly useful in dynamic healthcare environments like personalized treatment planning. Other types include semi-supervised learning, which combines labeled and unlabeled data, and self-supervised learning, often used for feature extraction and predictive modeling in healthcare applications. The specific tasks and applications of these types of ML, such as classification, clustering, disease diagnosis, and risk prediction as listed in **Table 1** demonstrate the wide-ranging impact of ML in healthcare, from prediction to patient segmentation.

LLMs are trained on massive text data using a phased approach that leverages the Transformer architecture. The Transformer's self-attention mechanism allows LLMs to efficiently process sequential data, enabling a strong grasp of context and meaning in text. Training LLMs begins with pretraining on large, unlabeled corpora (e.g., internet text, Wikipedia, social media) using a self-supervised approach 11 followed by fine-tuning on specific datasets with human feedback, tailoring the model to particular tasks. In the final phase, experts apply specialized prompting techniques to adapt the model for targeted applications, enabling it to handle nuanced, domain-specific tasks effectively. A prominent example of an LLM based on this Transformer architecture is OpenAI's ChatGPT 12, 13, which, after extensive training on diverse text sources, can generate coherent and contextually relevant responses across a wide range of topics.

## Applications of AI in CKD management

### Structure of the review

**Figure 2:**
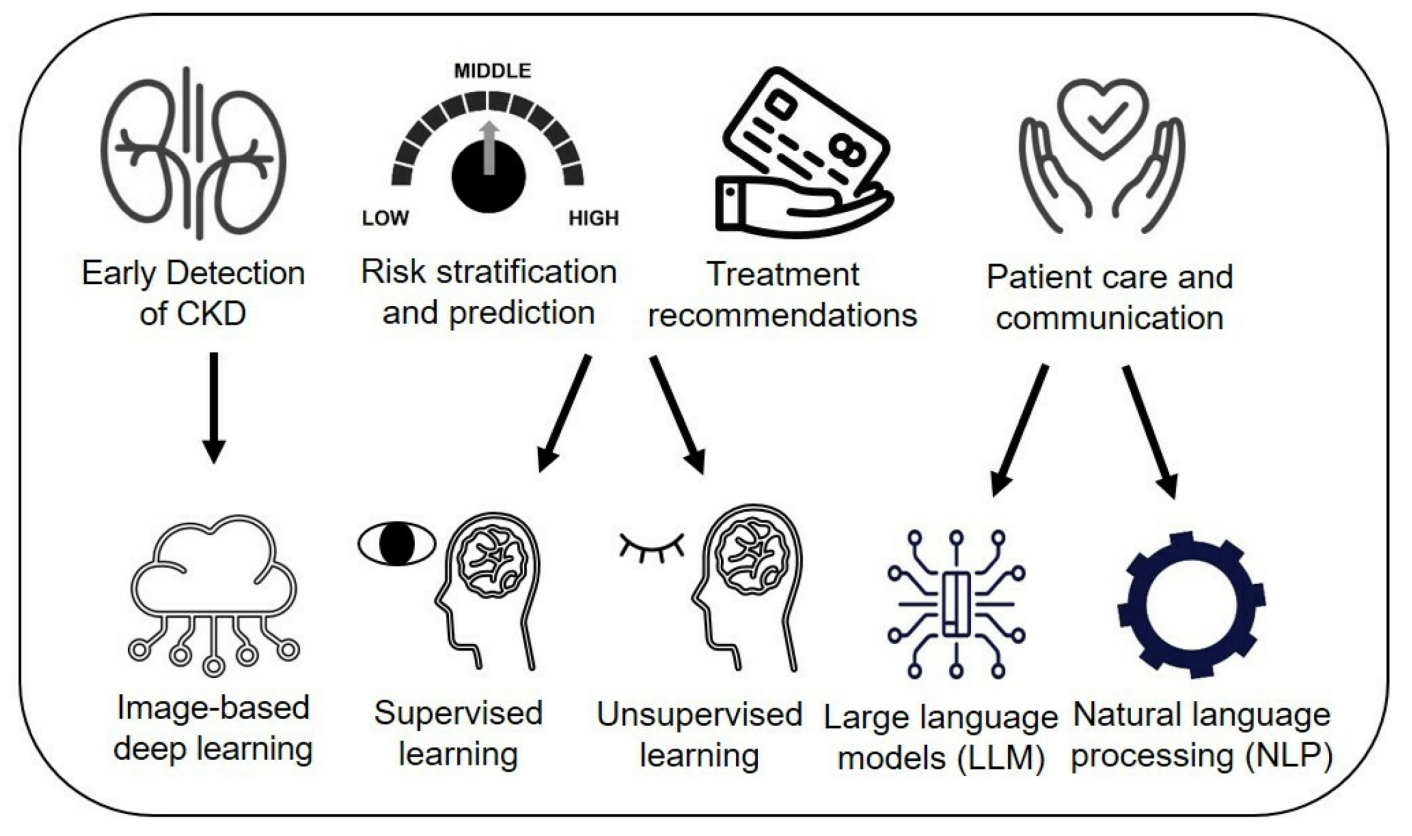
AI applications in CKD management.

We explored how AI contributes to CKD management across four areas (**Figure 2**): 1) early detection and screening, 2) risk stratification and prediction, 3) treatment recommendations and personalized care, and 4) patient care and communication. As previous reviews on AI in CKD have focused largely on traditional ML models for detecting or predicting CKD progression 8, 9, we excluded data-derived ML models for prognosis prediction but included them for risk stratification and treatment recommendations. Use cases include image-based DL techniques for detection, data-driven ML models that combine both supervised and unsupervised clustering for risk stratification, and prediction, and treatment planning through clinical decision support systems (CDSS) and NLP/LLM applications in enhancing patient communication and care. Additionally, we addressed AI challenges, such as ethical concerns, and proposed solutions, while exploring future directions like foundation models to create AI platforms supporting personalized care and patient self-management for CKD.

### Search strategy

We conducted a scoping review by identifying literature in PubMed from January 1, 2014, to September 30, 2024. The search was restricted to English language and adult studies, focusing on the past 10 years, which marked the period of DL 14 and when AI techniques began to significantly impact on medicine and nephrology. We excluded editorials, review articles, and articles related to specific kidney diseases other than CKD for e.g. acute renal failure, IgA nephropathy, polycystic kidney disease, diabetic nephropathy etc. We used search terms associated with CKD and AI models and their alternative terms. For e.g., for CKD, we used, end-stage kidney disease, renal failure, kidney failure, impaired kidney function, etc. We combined the search terms using Boolean Operators (“OR”, “AND”). We also reviewed the reference lists of the articles identified through our search strategy and selected additional articles that we deemed relevant. For our aim on image-based deep learning in CKD detection and screening, the search terms included “artificial intelligence” OR “deep learning algorithm” OR “neural networks” combined with “image” and CKD search terms. This search produced 28 articles, of which four were selected for inclusion and through back referencing, two more articles were included, resulting in a final reference list of six articles relevant to image-based deep learning. For risk stratification and prediction, we identified 14 articles, and for treatment recommendations, we found 4 articles. For our aim on patient care and communication using NLP and LLM, we identified 17 articles through direct searching, coauthor recommendations and back references. In total, we identified 41 use cases relevant to CKD management across various AI techniques which are discussed in the following sections.

## Early detection and screening

CKD often remains asymptomatic until advanced stages, but early detection through risk factor management can slow progression. Regular screening for CKD is recommended in the general population, and particularly for high-risk groups, such as those with diabetes, hypertension and certain ethnic groups since earlier detection enables timely interventions. Screening typically involves assessing estimated glomerular filtration rate (eGFR) or proteinuria. Despite the availability of effective treatments 15-19, CKD screening rates remain suboptimal, even in high-income countries and in those with diabetes. Serum creatinine, a key component in eGFR calculation, is influenced by factors such as muscle mass, diet and specific medication use, which can affect its precision 20. Similarly, albuminuria demonstrates significant intra-individual variability, with up to 50% fluctuation, and is also affected by physical activity and other factors. Additionally, up to 50% of individuals with diabetes and reduced eGFR may have albuminuria levels within the normal range 21, underscoring the limitations of current screening tools in detecting CKD in this population. The 24-hour urine collection is considered the gold standard for diagnosing microalbuminuria due to its minimal variability, but it is labor-intensive. In clinical practice, when CKD screening is recommended by physicians, the patient visits the laboratory on one day and is scheduled to return on another day for the physician to review the results which may involve multiple visits. Scaling up screening tests poses challenges due to limited resources. Deep learning algorithms (DLA) using noninvasive imaging, such as ultrasound kidney images or retinal images 22, may complement early CKD detection. Ultrasound-based parameters, such as kidney length, echogenicity, and cortical thickness, are influenced by CKD and offer a non-invasive means to assess renal function 23. Renal cortical echogenicity, being irreversible compared to serum creatinine fluctuations, serves as a stable marker of kidney health. However, substantial interobserver variability in ultrasound interpretation has historically limited its utility 24. Advances in deep learning for image segmentation and classification now enable standardized, objective analysis, enhancing diagnostic accuracy 25. Besides, ultrasound images, studies, including our own, have demonstrated that manually graded retinal features can predict CKD 26, 27, forming the basis for developing DLAs to detect CKD directly from retinal images. These imaging modalities offer accessible, cost-effective, and non-invasive options for screening, making them valuable tools for early CKD detection and management.

**Table 2** summarizes studies on image-based DLAs for CKD detection. Kuo *et al.* used over 4,500 kidney ultrasound images to predict eGFR and CKD, achieving an 85.6% accuracy, outperforming nephrologists (60.3%-80.1%) 28. However, lacking external validation limits its generalizability. We published two studies using retinal imaging to detect CKD in general 29 and diabetic populations^30^, later named RetiKid and RetiKid-Diab. RetiKid 29 achieved 91% accuracy in internal validation and 73%-83% in external validation across two populations, comparable to a traditional risk factor model including age, sex, ethnicity, diabetes and hypertension (92% internally, 83%-89% externally). RetiKid-Diab, developed for diabetic populations, achieved 83% accuracy internally and 73%-76% externally, performing similarly to risk factor models 30. Other studies, such as those by Kang *et al.*^31^ and Zhang *et al.*^32^ have also shown promising results for retinal image-based DLAs in CKD detection. An *et al.* used UK Biobank fundus images to train DLAs for detecting CKD (using creatinine-only vs. creatinine and cystatin-C eGFR) and chronic renal failure (ICD-10 codes) 33. DLAs trained on CKD defined by dual markers outperformed single markers (AUCs: 0.758 vs. 0.668, p<0.05), but performance for chronic renal failure was similar (AUCs: 0.712 vs. 0.690, p=0.1).

The advantages of using retinal images for CKD screening is that it is non-invasive, and retinal cameras which are commonly available in community and primary care settings for diabetic retinopathy (DR) screening can be utilized. Thus, it may prove useful in the following circumstances: First, in facilities with availability of retinal images from DR screening, CKD screening can be used as an 'add-on' to DR screening report using the same images. Second, non-invasive screening methods may be more tolerable for individuals with a fear of needles, potentially increasing participation rates. Fear of needles, which affects approximately 10% of individuals, is a common issue that can lead to the avoidance of medical care 34. Third, drawing blood may not be practical in all settings, for e.g. rural areas with lack of laboratory facilities. With improvement in imaging technologies and resolution of smart phones, RetiKid can be integrated into smartphones to give rapid, point-of-care diagnoses, which will help reduce demand on human resources involved in CKD screening services and could improve compliance to screening.

Finally, as RetiKid automates the screening process, it enables a larger number of at-risk patients, for e.g. those with family history of CKD to be screened more effectively. It will also allow screening to be done in a few minutes at any healthcare site or community site in the future, therefore increasing the scope of kidney screening to beyond the usual blood testing sites.

## Risk stratification and prediction

Risk stratification in CKD using ML involves two main approaches 35. First, predictive models utilizing supervised learning trained on electronic health records (EHR) or other patient data sources estimate individual risk of CKD progression and adverse outcomes. Second, unsupervised learning methods are employed to identify and characterize distinct sub-phenotypes of CKD, revealing underlying patterns and heterogeneity in the disease that can inform more personalized treatment strategies.

### Using supervised learning

Two validated ML-based risk scores, KidneyIntelX 36 and Klinrisk 37 stratify patients with preserved kidney function (eGFR ≥60) to identify those at high risk of CKD progression. KidneyIntelX developed by Chan *et al.* uses KDIGO components, clinical variables, and three blood-based biomarkers to categorize patients into low, intermediate, and high risk, with high-risk patients 2.5 times more likely to be referred to specialty care 36. It is also the first FDA-authorized test for assessing CKD progression risk in adults with type 2 diabetes. In the CANVAS trial, KidneyIntelX effectively stratified participants with baseline DKD into low- (42%), intermediate- (44%), and high-risk (15%) groups, with cumulative incidence rates of 3%, 11%, and 26%, respectively 38. Klinrisk, developed by Ferguson *et al.*, uses a random forest model to risk stratify individuals, predicting that the top 30% of those classified as high- and intermediate-risk account for 87% of progression events over 2 years and 77% over 5 years 37.

A US study used convolutional neural networks (CNN) models trained on kidney biopsy images to classify tasks including CKD severity (multi-class label), baseline serum creatinine and nephrotic-range proteinuria at the time of biopsy (binarized values), and predicting renal survival at 1-, 3-, and 5-years and compared the performance of CNN models to pathologist-estimated fibrosis score (PEFS) 39. The CNN achieved superior accuracy for all outcomes compared to PEFS (Kappa 0.519 vs. 0.051 for CKD staging; accuracy of 91% vs. 84% for serum creatinine, 87% vs. 70% for nephrotic-range proteinuria, and 88%, 88% and 90% vs. 81%, 80% and 79% for predicting 1-, 3-, and 5-year renal survival). With further validation across diverse clinical practices and image datasets, this AI tool has the potential to be deployed at the point of care, enhancing clinical decision-making for nephrologists.

### Using unsupervised clustering

Inaguma *et al.* used hierarchical clustering to identify eGFR trajectories and a random forest (RF) model to predict CKD progression (eGFR decline ≥30% within 2 years), achieving 79% accuracy for rapid eGFR decline, with hemoglobin, albumin, and CRP as key predictors 40. Kaufmann *et al.* used K-Means clustering to identify four patient groups with varying eGFR trajectories before dialysis, finding higher hospitalization and mortality risks in those with rapid eGFR decline 41. The Chronic Renal Insufficiency Cohort (CRIC) study applied consensus clustering to categorize CKD patients into three clusters based on 72 characteristics, revealing significant associations with cardiovascular disease and mortality, although cluster membership did not improve risk prediction beyond traditional markers like eGFR and albuminuria 42. Using time-series clustering, Saito *et al.* stratified CKD patients (baseline eGFR ≥45 mL/min/1.73m²) into five groups based on 5-year eGFR changes, ranging from 4.9% to 45.1% decline 43. A prediction model with light gradient boosting achieved 68% accuracy overall and 76% for predicting severe decline (Classes 4 and 5). SHapley Additive exPlanations (SHAP) analysis highlighted baseline eGFR (1.61), hemoglobin (0.12), and BMI (0.11) as key contributors. While clustering did not improve prediction accuracy, these studies demonstrate the utility of unsupervised algorithms for phenotyping and exploring CKD heterogeneity.

### Using digital twin technology

Digital twins are virtual replicas or models of individual patients, crafted to mirror their physiological characteristics, health status, and disease progression in real time 44. Powered by data from various sources such as EHR, lab reports, imaging reports, genetics, wearable devices etc. AI algorithms create sophisticated computational models that simulate a patient's biological processes. Just like a flight simulator helps pilots practice and understand different flight scenarios, digital twin models allow doctors to simulate and analyze how various factors, such as lifestyle changes, medications, or genetic mutations affect disease progression leading to more personalized treatment plans.

Researchers in Singapore developed a digital twin model using generalized metabolic fluxes (GMF) to predict CKD onset within three years across four multi-ethnic cohorts (US and Singapore) 45. The prediction model reached 75-86%, revealing worse metabolic profiles in future CKD patients. Participants were stratified into high-, moderate-, and low-risk groups, with CKD development rates of 53.9-62.9%, 17.3-19.3%, and 5.4-10.7%, respectively. GMF combined with K-means clustering reveal distinct metabolic profiles driving CKD progression, with future CKD patients exhibiting elevated fluxes linked to circulation, blood pressure, glucose metabolism, and kidney function. This approach maps patient health trajectories, enabling risk stratification, sub-grouping, and clustering based on metabolic health.

### CKD and CVD risk

Apart from CKD applications, few studies have explored the use of ML models for assessing CVD risk in CKD. A recent cross-sectional study in China, using the random forest algorithm, identified age, systolic blood pressure, and sublingual microcirculatory perfusion parameters (total and perfused vessel density, TVD and PVD) as optimal predictors for cardiovascular-kidney-metabolic (CKM) risk in type 2 diabetes mellitus (T2DM) patients 46. Another study in China developed CVD risk prediction models for patients with CKD using routine clinical diagnostic and treatment data extracted from EMR 47. By applying Least Absolute Shrinkage and Selection Operator (LASSO) regression, the study identified eight critical predictors of CVD risk, including age, history of hypertension, high-density lipoprotein levels, and urinary protein. Among various ML approaches tested, the extreme gradient boosting model demonstrated superior predictive performance with accuracy of 89%. These findings underscore the potential of ML models in cardiovascular risk stratification among CKD patients, offering opportunities to enhance CKD management strategies. However, external validation is necessary to confirm their broader applicability and usefulness.

## Treatment recommendations and personalized management

Recent advancements in CDSS have enabled personalized management of CKD. Anemia in CKD patients remains a significant risk factor despite advances in dialysis, erythropoietin stimulating agents (ESAs), and injectable iron therapies 48. Barbieri *et al.* developed a data-driven ML model the 'Anemia Control Model (ACM)' to generate individualized ESA dose recommendations based on patient history, dose-response information, and demographic data, supporting anemia management in hemodialysis patients 49. The ACM incorporates an ANN and a dose selection algorithm to optimize ESA dosing. The ANN, trained on 170,000 clinical records and tested on 40,000, generated optimal darbepoetin and iron doses to achieve target Hb and ferritin levels. Deployment of the ACM in 752 patients undergoing hemodialysis therapy in three pilot clinics, reduced ESA use, minimized Hb fluctuations, and improved anemia management. A subsequent prospective study in Spain validated the model's effectiveness, showing reduced cardiovascular events, transfusions, hospitalization, and costs, enhancing patient care 50. This AI-driven CDSS has the potential to improve prescribing practices that typically react excessively to Hb fluctuations, ultimately enhancing patient care and treatment outcomes.

Li *et al.* developed TrajVis, a web-based interactive visual CDSS designed to visualize AI-driven CKD patient trajectories. This tool aids clinicians in understanding CKD progression by leveraging the DisEase PrOgression Trajectory (DEPOT) model, a graph-based AI approach that uses latent representation to infer disease trajectories and support personalized patient management 51. TrajVis features four panels: Patient View (demographics and clinical data), Trajectory View (visualizing CKD trajectories), Clinical Indicator View (longitudinal patterns), and Analysis View (individual progression trajectories). Using synthetic EHR data, the web app helps clinicians summarize data, visualize progression, and identify risk factors. TrajVis complements KFRE by incorporating comorbidities, past patterns, and treatment adherence, providing tailored predictions to support nephrologists in managing CKD progression.

Kalmrowski *et al.* utilised random forest model to predict the risk of kidney failure in patients with advanced CKD over 6- and 12-month time frames 52 using patient data, such as age, sex, and time-varying trends in laboratory measurements. The model accurately identified unplanned dialysis in 88% of cases at 6 months and 87% at 12 months, with external validation confirming 87% accuracy at both timepoints. This early warning system aids clinical decision-making, enabling timely interventions and smoother transitions to kidney failure care at both timepoints.

## Patient care and communication

NLP has emerged as a powerful tool in CKD management, with various use cases aimed at improving documentation, symptom detection, data extraction, and disease tracking from unstructured clinical notes and reports.

### Identification of disease/patient phenotypes

Two NLP-based tools developed to assess CKD documentation in EHRs, achieved 95.4%-99.8% sensitivity and 99.8% specificity 53. Analysis revealed 22% of moderate CKD cases were undocumented, potentially delaying treatment. NLP was used to detect seven hemodialysis symptoms, showing higher sensitivity (0.85-0.99) than ICD codes (0.09-0.59) 54. In Canada, an NLP system identified dementia (99%), diabetes (83%), and infarction (80%) from dialysis notes 55. Another Canadian study applied NLP to kidney biopsy reports, creating a structured database linked to clinical data, improving disease classification and patient management 56.

### Enhancing risk prediction models

NLP has been used to extract concepts from unstructured clinical notes to identify predictors of CKD progression, such as high-dose ascorbic acid and fast-food consumption 57. Latent Dirichlet Allocation, an unsupervised ML technique, uncovered themes related to diabetes, including insulin and glucose, which were linked to a higher risk of progression from CKD stage 3 to stage 4 58. Models combining longitudinal lab data with NLP-extracted clinical text achieved 85% accuracy in predicting progression to stage 4, outperforming models based on lab results alone (82%) or eGFR (78%).

These capabilities highlight NLP's potential to improve CKD care by detecting undocumented cases, identifying early signs of progression, and supporting personalized treatment and proactive population health management. While NLP has shown promise for improved patient care, its clinical application remains limited due to the need for external validation across different healthcare systems, complicated by unique note templates, settings-related and regional variations. Sharing progress notes for validation is challenging due to the presence of protected health information (PHI) and strict privacy protections, though automated deidentification solutions 59 offer potential to overcome these barriers.

### Improving patient care using LLMs

AI tools, particularly LLMs like ChatGPT/GPT-4, are revolutionizing patient care by streamlining various tasks 60. Physician burnout, affecting 63% of U.S. physicians 61, is a major issue that impacts healthcare efficiency, leading to more errors, lower patient satisfaction, and higher turnover. A key contributor to burnout is the excessive workload, including medical documentation and communication. AI, especially LLMs, can alleviate this by generating accurate discharge summaries, operative reports, and informed consent documents, sometimes outperforming traditional methods. Despite occasional inaccuracies, ChatGPT's potential to improve documentation quality and empathy in patient communication makes it a promising tool for reducing burnout. Specific use cases and examples of using LLMs, particularly ChatGPT, in patient care are detailed below:

### AI/digital scribes

The digital scribe uses automatic speech recognition (ASR) and NLP to automate clinical documentation, including creating notes, adding billing codes, and supporting diagnoses 62. This reduces physician documentation burden, enhances accuracy, and supports clinical decision-making. A proof-of-concept system developed by Klan *et al.* demonstrated ASR's feasibility for single-speaker physician-patient encounters 63. Expanding this, an Intelligent Listening Framework (ILF) was developed for multi-speaker environments, integrating advanced NLP techniques to capture and structure doctor-patient interactions.

In a study including 100 simulated nephrology cases, ChatGPT-4.0 outperformed ChatGPT-3.5 in identifying ICD-10 codes for nephrology conditions, with accuracy rates of 99% compared to 91% with consistent performance across two rounds 64. While ChatGPT-4.0 showed promise in reducing physician workload, the study highlights the need for further refinement, including multi-code generation and EHR integration.

### Patient messaging

Efficiently managing EHR inbox messages has become a significant challenge for healthcare providers, contributing to clinician burnout. Pham *et al.* assessed ChatGPT-4's performance in triaging simulated nephrology patient messages as non-urgent, urgent, or emergent 65. In two trials with 150 messages, ChatGPT-4 correctly categorized 93% of the messages, with minor overestimation (3-6%) and underestimation (1-4%). The system showed high internal consistency (Kappa score of 0.88), indicating that AI-driven triage could improve efficiency and patient care in outpatient nephrology settings.

### Patient education

LLMs support patient education by providing tailored materials and answering questions in understandable language, helping patients better manage chronic diseases and adhere to therapies 66. ChatGPT has been shown to generate recommendations for cardiovascular disease prevention 67, breast cancer screening and prevention 68 and kidney cancer 69.

### Clinical decision support

ChatGPT offers personalized dietary advice and assists clinicians with diagnosis and treatment recommendations by analyzing patient data against medical guidelines 70. Using a retrieval-augmented generation (RAG) method, models like ChatGPT can access real-time external sources, aligning with updated guidelines, such as the KDIGO 2023 CKD guidelines, ensuring accurate, current recommendations while reducing hallucination issues 71.

Medication management in nephrology is complex, and ChatGPT can assist by checking drug interactions, adjusting dosages based on renal function, and monitoring adherence 66. A study found ChatGPT-4 accurately identified 88% of 25 nephrology medications from self-captured pill images, with misidentifications mainly due to imprint issues 72. Despite this, it showed improved accuracy after feedback, highlighting its potential for medication identification. ChatGPT has also demonstrated value in clinical decision-making, such as identifying treatment-resistant schizophrenia 73 and suggesting treatment plans aligned with medical standards.

### Improving provider-patient communication

ChatGPT can assist in drafting correspondence, such as patient letters 74, and improving communication between healthcare professionals and patients about medication-related queries and side effects. Ayers *et al.* evaluated ChatGPT's performance using a public database from Reddit's r/AskDocs, analyzing 195 exchanges with verified physician responses 75. ChatGPT's responses were preferred 78.6% of the time, with higher ratings for quality and empathy. These findings suggest AI chatbots could ease clinician workload and reduce burnout 75. Incorporating prompt engineering (PE) into LLMs significantly enhances output accuracy by optimizing question framing and interpretation.

LLMs also provide fast, accurate translations in multiple languages, aiding clinical decision-making and improving therapy adherence. A recent study on ChatGPT's translation of 54 kidney transplant FAQs from English to Spanish found both GPT-3.5 and GPT-4.0 translations to be linguistically accurate and culturally sensitive, improving access to medical information for non-English-speaking populations 76.

### Administrative efficiency

ChatGPT can streamline administrative tasks by automating appointment scheduling, reminders, discharge summaries, and billing codes. It also helps patients track symptoms and medication adherence with automated reminders. This reduces the administrative burden on healthcare staff, freeing up time for direct patient care and improving healthcare efficiency. A study on ChatGPT's ability to generate a history of present illness showed that iterative PE improved its accuracy from 10.0% to 43.3%, matching the performance of senior medical residents 77. ChatGPT has also proven successful in generating accurate discharge summaries and operative reports, with AI-created visuals enhancing the quality of these notes 78.

## Challenges and future directions

AI has the potential to optimize CKD management by uncovering complex disease patterns and offering predictive insights, such as early detection, risk identification, and advanced image analysis 4. Reinforcement learning may further advance AI by providing real-time treatment recommendations.

However, integrating AI models into healthcare presents significant challenges, including technical hurdles related to data quality and access, model accuracy, interpretability, as well as adoption issues such as workflow integration, user training, and regulatory approval (**Table 3**).

### Technical challenges

**Data quality and access**: AI systems depend on reliable and valid training datasets, which are often difficult to procure. Access to high-quality data can be limited due to varying levels of digitization across healthcare institutions and restrictive data-sharing policies. Data sharing across institutions is crucial for developing more generalizable and robust models, but direct data sharing poses privacy concerns and logistical challenges. Federated learning offers an alternative approach, allowing collaborative model development without directly sharing patient data. In this method, local models are trained independently at each institution, and only the gradients or coefficients are sent to a centralized global model. This ensures privacy by design while integrating knowledge from multiple institutions, which is essential for developing AI-enabled decision support systems.

**Bias:** Algorithmic bias occurs when an algorithm produces skewed outcomes due to biased training data or flawed design, leading to inaccurate predictions for underrepresented groups. For example, models may underperform for minority populations or younger patients if trained on data skewed toward older or majority groups. To address this, ensuring diverse data and regular auditing of algorithm outputs is crucial. Integrating AI into clinical workflows ensures its actionability and effectiveness in real-time practice 79.

**Model accuracy:** Modest predictive accuracy and lack of clinical utility are challenges for image-based DL models. Future research should focus on standardizing image acquisition, establishing multicenter data-sharing platforms, and exploring diverse biomarkers 80. Combining multimodal imaging with clinical data shows promise for improving model performance and clinical relevance 81.

Specifically in diagnosis, CKD poses significant challenges in the collection of imaging and pathological data, as only a small subset of patients undergo renal biopsy, and the limited tissue obtained is often insufficient for advanced sequencing. ML models leveraging multimodal frameworks can help overcome these limitations by integrating diverse data sources, including unstructured health records, pathological findings, imaging modalities, Omics, laboratory results, and clinical biomarkers to enhance predictive accuracy. Techniques like transfer learning and few-shot learning can optimize performance with small datasets, while generative adversarial networks (GANs) can generate synthetic data to augment training sets without compromising privacy. Additionally, temporal ML models incorporating longitudinal data, such as changes in imaging features and clinical parameters, hold promise for predicting treatment responses and recovery trajectories.

In medical image analysis, foundation models enhance AI by offering a pre-trained base for specialized models, improving tasks like image classification. They support zero-shot learning, where the model is applied to a test set without task-specific training, and few-shot learning, where a small number of labeled examples are used to adapt to a task. Pre-trained on diverse datasets, these models capture broad patterns and knowledge, improving performance and reducing the need for extensive task-specific training 82. RETFound, a retinal image-based foundation model, was pre-trained on 1.6 million unlabeled fundus photographs using self-supervised learning. It achieved higher performance than comparison models with fewer labeled data, enabling effective diagnosis and prognosis of sight-threatening eye diseases and predicting heart failure, acute myocardial infarction (AMI), and stroke 83. The trend in image analysis is moving towards foundation models, with open-source models like RETFound allowing for the creation of more effective models with less data.

Interpretability of AI models is crucial for patient care, as understanding a model's reasoning can impact decisions. For image-based deep learning models, techniques like heat maps or saliency maps highlight key influencing areas. In LLMs, attention mechanisms show which parts of the input the model focuses on, while Layer-Wise Relevance Propagation (LRP) traces each input feature's contribution. Model probing analyzes the model's internal workings to reveal decision-making processes.

Hallucinations in LLMs occur when models generate inaccurate or fabricated information, undermining trust in their predictions 11. To mitigate this, PE can refine inputs for more accurate outputs 84, 85. Additionally, domain-specific models, like BioMedLM 2.7B, ClinicalBERT, and Med-PaLM2, are fine-tuned on specialized datasets to improve accuracy in medical fields 11, 86. The RAG framework enhances LLMs by integrating external knowledge bases, increasing accuracy, explainability, and transparency. This integration allows healthcare systems to use proprietary hospital data, ensuring functionality and Health Insurance Portability and Accountability Act (HIPAA), compliance while maintaining patient privacy.

**Non-technical challenges**: Ethical concerns around data privacy and security demand robust systems for managing data collection, storage, and sharing while establishing clear guidelines for patient autonomy and privacy. Financially, the substantial costs associated with AI implementation require investments in technology, infrastructure, and user training. To mitigate these costs, fostering a collaborative ecosystem among healthcare professionals, researchers, regulators, and industries can optimize resource use. Regulatory approval is essential for medical devices using AI algorithms in clinical decision-making, but navigating these frameworks is often complex and time-consuming. Streamlining the approval process is crucial to overcoming regulatory hurdles for AI-based medical devices. Specifically, there is currently no established regulatory framework for integrating LLM-based devices into clinical use, though this landscape is rapidly evolving. It is crucial for researchers and developers to recognize the importance of building these models with a Quality Management System (QMS) in place, along with a plan for external validation and managing future changes. Legal issues in using AI technologies include cybersecurity threats such as data breach, unauthorized access, or liability concerns due to incorrect recommendations provided by AI tools that may impact patient care. Strong data encryption and clear accountability frameworks are needed to safeguard patient information and clarify liability for AI errors. Collaboration among healthcare providers, researchers, regulators, and industries is essential to develop robust protocols, ensuring compliance with legal standards while minimizing risks and maintaining patient safety. By addressing these key areas, the barriers for successful implementation can be overcome to pave the way for effective AI integration within healthcare systems.

## Figures and Tables

**Table 1:** Overview of machine learning types, tasks, and applications in healthcare

Types of ML	Description	Tasks	Applications
Supervised learning	Machines are trained on labeled datasets where input (features) and output (labels) are already mapped	• Classification: To predict a categorical variable • Regression: To predict a continuous variable	• Diagnosis, risk stratification • Risk prediction
Unsupervised learning	Machines are trained on unlabeled datasets. The algorithm identifies patterns in the unlabeled dataset without explicit instructions	• Clustering: Grouping similar data points • Anomaly detection: Identifies unusual patterns or outliers in data	• Patient segmentation or disease patterns • Finding subtle changes in patient data that might signal potential complications
Semi-supervised learning	Combines small amounts of labeled data with large amounts of unlabeled data	• Classification • Clustering	• Medical image analysis with limited labels
Reinforcement learning	Learns to make decisions through trial and error to maximize rewards	• Sequential decision making	• Dose optimisation, personalized treatment recommendations
Self-supervised learning	Learns to predict part of the data from other parts, often used for representation learning	• Feature extraction • Pretext tasks	• Representation learning • Predictive modeling
Deep learning (DL)	Uses multi-layered neural networks to model complex patterns in large datasets	• Image recognition • Natural language processing	• Retinal image analysis • Chatbots for patient care

**Table 2:** Image-based deep learning algorithms for detecting CKD

Publication reference	Study population	Input	DL architecture	Outcome	Performance metrics	
					Internal validation	External validation
Kuo CC *et al.*, 2019^28^	1,299 patients, aged 18-89 years enrolled in Taiwan's National Health Insurance	4,505 kidney ultrasound images	Transfer learning with ResNet model pretrained on ImageNet dataset	eGFR level and CKD status eGFR<60	Predicting eGFR: PCC: 0.741 and MAE:17.6 CKD status accuracy: 85.6% (nephrologists' 60.3%-80.1%)	Not done
Sabanayagam C *et al.*, 2020^29^	Development cohort, SEED study, n = 1297 participants, 40-80 y. External: SP2 study, n = 3735 aged ≥25 y. BES study, n = 1538 aged ≥40 y	1) Retinal images (n = 12,970 for SEED, 7470 for SP2, 3076 for BES) 2) Risk factor (RF) model	Convolutional neural network (cCondenseNet)	CKD (eGFR<60) status	AUC (image only): 0.911 in SEED AUC (RF): 0.916 in SEED	AUC (image only): SP2, 0.733 BES, 0.835 AUC (RF): SP2, 0.829 BES, 0.887
Kang EY *et al.*, 2020^31^	Development cohort: 6212 patients in a hospital in Taiwan who underwent retinal imaging and laboratory tests	25,706 retinal images	VGG-19 CNN	Early renal function impairment (eGFR <90 mL/min/1.73 m^2^)	AUC= 0.81	Not done
Zhang K *et al.*, 2021^32^	Development cohort: 43156 participants from a cross-sectional database (CC-FII) External: 8,059 individuals from Guangdong Province	1. Retinal fundus images (n = 86,312) 2. Metadata 3. Combined	Convolutional Neural Network (CNN)	1. CKD stage 1-5 2. eGFR prediction	1. CKD detection: Image only: 0.923 Metadata: 0.828 Combined: 0.929 2. eGFR prediction PCC=0.716 MAE=11.1	1. CKD detection: Image only: 0.854 Metadata: 0.796 Combined: 0.871 2. eGFR prediction PCC=0.700 MAE=12.9
Betzler BK *et al.*, 2023^30^	Development cohort: 6066 primary care-based participants with diabetes (SiDRP) External-1: 1885 population-based participants with diabetes (SEED) 2. External-II, 439 clinic-based participants with diabetes (SMART2D)	1. Retinal fundus images 2. RF 3. Hybrid	cCondenseNet	Diabetic kidney disease (DKD) defined as eGFR<60	AUC in SiDRP Image only: 0.826 RF: 0.847 Hybrid: 0.866	AUC (image only): SEED: 0.764 SMART2D: 0.726 AUC, RF only SEED: 0.802 SMART2D: 0.701 AUC, Hybrid SEED: 0.828 SMART2D: 0.761
An S *et al.*, 2023^33^	12636 adults from the UK Biobank.	Retinal images (n = 14040)	Ensemble models based on ResNet-50, EfficientNetV2	CKD (eGFR<60) 1) defined using serum creatinine based eGFR (Cr-eGFR) 2) Creatinine + Cystatin C-based eGFR (Cr+Cys-eGFR) 2) Chronic renal failure (CRF) from ICD-10 codes	AUC for CKD 0.668 for Cr-eGFR 0.758 for Cr+Cys-eGFR (p<0.01) AUC for CRF 0.690 for Cr-eGFR 0.712 for Cr+Cys-eGFR p=0.1	Not done

Abbreviations: AUC, Area under curve; BES, Beijing Eye Study; CKD, chronic kidney disease; MAE, mean absolute error; PCC, Pearson's correlation coefficient; SEED, ​Singapore Epidemiology Of Eye Diseases; SIDRP, Singapore Integrated Diabetic Retinopathy Programme; SMART2D, Singapore Study of Macro-Angiopathy and microvascular Reactivity in Type 2 Diabetes; SP2, Singapore Prospective Study Program 2

**Table 3:** Technical and non-technical challenges of using AI models and potential solutions to

Technical challenges	Solutions
Data quality and data access	Develop and implement secure data collection, storage, and sharing systems across institutions for generalizing models and enhancing their applicability to diverse patient populations. Federated learning allowing collaborative model training on decentralized data while preserving patient privacy
Bias (Algorithmic and indication)	Algorithmic bias: Models should include diverse data and adequate representation of minority groups Bias by indication: Models should utilize routinely collected data from standard care and AI algorithms to be integrated into clinical workflow
Model accuracy	Multimodal approaches by integrating unstructured electronic health records (EHRs) with time series, imaging, Omics data, lab results, and other data sources. Foundation models provide a pretrained base for specialized models which can be fine-tuned for specific tasks with smaller labeled datasets.
Model interpretability	Employ explainable AI techniques such as LIME, SHAP, and saliency maps for ML/DL models Attention mechanisms, model probing etc. for LLMs
Hallucinations (LLM)	Prompt engineering (PE) to provide more accurate outputs Using domain-specific models which are fine-tuned on specialized datasets
Non-technical challenges	
Ethical concerns	Clear guidelines for patient autonomy and privacy
Financial cost	Fostering a collaborative ecosystem among healthcare professionals, researchers, regulators, and industries to optimize resource use Invest in training healthcare professionals on AI concepts and applications
Regulatory hurdles	Streamline approval processes for AI-based medical devices
Cybersecurity threats	Implementing robust security protocols Developing comprehensive legal frameworks.

Abbreviations: AI, artificial intelligence; DL, deep learning; LIME, local interpretable model-agnostic explanations; LLMs, large language models; ML, machine learning; SHAP, SHapley Additive exPlanations;

## Abbreviations

AI: artificial intelligence
ACM: Anemia Control Model
AKI: acute kidney injury
AMI: acute myocardial infarction
ANN: artificial neural networks
ASR: automatic speech recognition
CDSS: clinical decision support systems
CKD: chronic kidney disease
CNN: convolutional neural networks
CRIC: Chronic Renal Insufficiency Cohort
CVD: cardiovascular disease
DKD: diabetic kidney disease
DLA: deep learning algorithms
DL: deep learning
DR: diabetic retinopathy
eGFR: estimated glomerular filtration rate
ESAs: erythropoietin stimulating agents
GMF: generalized metabolic fluxes
EHR: electronic health records
HIPAA: Health Insurance Portability and Accountability Act
ILF: intelligent listening framework
ML: machine learning
NLP: natural language processing
LLMs: large language models
LRP: layer-wise relevance propagation
PEFS: pathologist-estimated fibrosis score
PE: prompt engineering
PHI: protected health information
QMS: quality management system
RF: random forest
SHAP: SHapley Additive exPlanations

## References

[1] Hill NR, Fatoba ST, Oke JL, Hirst JA, O'Callaghan CA, Lasserson DS (2016). Global Prevalence of Chronic Kidney Disease - A Systematic Review and Meta-Analysis. PLoS One.

[2] Foreman KJ, Marquez N, Dolgert A, Fukutaki K, Fullman N, McGaughey M (2018). Forecasting life expectancy, years of life lost, and all-cause and cause-specific mortality for 250 causes of death: reference and alternative scenarios for 2016-40 for 195 countries and territories. Lancet.

[3] Collaboration GBDCKD (2020). Global, regional, and national burden of chronic kidney disease, 1990-2017: a systematic analysis for the Global Burden of Disease Study 2017. Lancet.

[4] Loftus TJ, Shickel B, Ozrazgat-Baslanti T, Ren Y, Glicksberg BS, Cao J (2022). Artificial intelligence-enabled decision support in nephrology. Nat Rev Nephrol.

[5] Yuan Q, Zhang H, Deng T, Tang S, Yuan X, Tang W (2020). Role of Artificial Intelligence in Kidney Disease. Int J Med Sci.

[6] Burlacu A, Iftene A, Jugrin D, Popa IV, Lupu PM, Vlad C (2020). Using Artificial Intelligence Resources in Dialysis and Kidney Transplant Patients: A Literature Review. Biomed Res Int.

[7] Thongprayoon C, Kaewput W, Kovvuru K, Hansrivijit P, Kanduri SR, Bathini T (2020). Promises of Big Data and Artificial Intelligence in Nephrology and Transplantation. J Clin Med.

[8] Lei N, Zhang X, Wei M, Lao B, Xu X, Zhang M (2022). Machine learning algorithms' accuracy in predicting kidney disease progression: a systematic review and meta-analysis. BMC Med Inform Decis Mak.

[9] Sanmarchi F, Fanconi C, Golinelli D, Gori D, Hernandez-Boussard T, Capodici A (2023). Predict, diagnose, and treat chronic kidney disease with machine learning: a systematic literature review. J Nephrol.

[10] Farrell D, Chan L (2023). Application of Natural Language Processing in Nephrology Research. Clin J Am Soc Nephrol.

[11] Omiye JA, Gui H, Rezaei SJ, Zou J, Daneshjou R (2024). Large Language Models in Medicine: The Potentials and Pitfalls: A Narrative Review. Ann Intern Med.

[12] Thirunavukarasu AJ, Ting DSJ, Elangovan K, Gutierrez L, Tan TF, Ting DSW (2023). Large language models in medicine. Nat Med.

[13] Betzler BK, Chen H, Cheng CY, Lee CS, Ning G, Song SJ (2023). Large language models and their impact in ophthalmology. Lancet Digit Health.

[14] LeCun Y, Bengio Y, Hinton G (2015). Deep learning. Nature.

[15] Heerspink HJL, Stefansson BV, Correa-Rotter R, Chertow GM, Greene T, Hou FF (2020). Dapagliflozin in Patients with Chronic Kidney Disease. N Engl J Med.

[16] The E-KCG, Herrington WG, Staplin N, Wanner C, Green JB, Hauske SJ (2023). Empagliflozin in Patients with Chronic Kidney Disease. N Engl J Med.

[17] Pitt B, Filippatos G, Agarwal R, Anker SD, Bakris GL, Rossing P (2021). Cardiovascular Events with Finerenone in Kidney Disease and Type 2 Diabetes. N Engl J Med.

[18] Perkovic V, Tuttle KR, Rossing P, Mahaffey KW, Mann JFE, Bakris G (2024). Effects of Semaglutide on Chronic Kidney Disease in Patients with Type 2 Diabetes. N Engl J Med.

[19] Keane WF, Lyle PA, Reduction of Endpoints in NwtAIIRALs (2003). Recent advances in management of type 2 diabetes and nephropathy: lessons from the RENAAL study. Am J Kidney Dis.

[20] Kidney Disease (2024). Improving Global Outcomes CKDWG. KDIGO 2024 Clinical Practice Guideline for the Evaluation and Management of Chronic Kidney Disease. Kidney Int.

[21] Mottl AK, Kwon KS, Mauer M, Mayer-Davis EJ, Hogan SL, Kshirsagar AV (2013). Normoalbuminuric diabetic kidney disease in the U.S. population. J Diabetes Complications.

[22] Grzybowski A, Jin K, Zhou J, Pan X, Wang M, Ye J (2024). Retina Fundus Photograph-Based Artificial Intelligence Algorithms in Medicine: A Systematic Review. Ophthalmol Ther.

[23] O'Neill WC (2000). Sonographic evaluation of renal failure. Am J Kidney Dis.

[24] Yaprak M, Cakir O, Turan MN, Dayanan R, Akin S, Degirmen E (2017). Role of ultrasonographic chronic kidney disease score in the assessment of chronic kidney disease. Int Urol Nephrol.

[25] Shen D, Wu G, Suk HI (2017). Deep Learning in Medical Image Analysis. Annu Rev Biomed Eng.

[26] Wong CW, Wong TY, Cheng CY, Sabanayagam C (2014). Kidney and eye diseases: common risk factors, etiological mechanisms, and pathways. Kidney Int.

[27] Yip W, Ong PG, Teo BW, Cheung CY, Tai ES, Cheng CY (2017). Retinal Vascular Imaging Markers and Incident Chronic Kidney Disease: A Prospective Cohort Study. Sci Rep.

[28] Kuo CC, Chang CM, Liu KT, Lin WK, Chiang HY, Chung CW (2019). Automation of the kidney function prediction and classification through ultrasound-based kidney imaging using deep learning. NPJ Digit Med.

[29] Sabanayagam C, Xu D, Ting DSW, Nusinovici S, Banu R, Hamzah H (2020). A deep learning algorithm to detect chronic kidney disease from retinal photographs in community-based populations. Lancet Digit Health.

[30] Betzler BK, Chee EYL, He F, Lim CC, Ho J, Hamzah H (2023). Deep learning algorithms to detect diabetic kidney disease from retinal photographs in multiethnic populations with diabetes. J Am Med Inform Assoc.

[31] Kang EY, Hsieh YT, Li CH, Huang YJ, Kuo CF, Kang JH (2020). Deep Learning-Based Detection of Early Renal Function Impairment Using Retinal Fundus Images: Model Development and Validation. JMIR Med Inform.

[32] Zhang K, Liu X, Xu J, Yuan J, Cai W, Chen T (2021). Deep-learning models for the detection and incidence prediction of chronic kidney disease and type 2 diabetes from retinal fundus images. Nat Biomed Eng.

[33] An S, Vaghefi E, Yang S, Xie L, Squirrell D (2023). Examination of alternative eGFR definitions on the performance of deep learning models for detection of chronic kidney disease from fundus photographs. PLoS One.

[34] Deacon B, Abramowitz J (2006). Fear of needles and vasovagal reactions among phlebotomy patients. J Anxiety Disord.

[35] Pereira-Morales AJ, Rojas LH (2022). Risk stratification using Artificial Intelligence: Could it be useful to reduce the burden of chronic kidney disease in low- and middle-income Countries?. Front Public Health.

[36] Chan L, Nadkarni GN, Fleming F, McCullough JR, Connolly P, Mosoyan G (2021). Derivation and validation of a machine learning risk score using biomarker and electronic patient data to predict progression of diabetic kidney disease. Diabetologia.

[37] Ferguson T, Ravani P, Sood MM, Clarke A, Komenda P, Rigatto C (2022). Development and External Validation of a Machine Learning Model for Progression of CKD. Kidney Int Rep.

[38] Lam D, Nadkarni GN, Mosoyan G, Neal B, Mahaffey KW, Rosenthal N (2022). Clinical Utility of KidneyIntelX in Early Stages of Diabetic Kidney Disease in the CANVAS Trial. Am J Nephrol.

[39] Kolachalama VB, Singh P, Lin CQ, Mun D, Belghasem ME, Henderson JM (2018). Association of Pathological Fibrosis With Renal Survival Using Deep Neural Networks. Kidney Int Rep.

[40] Inaguma D, Hayashi H, Yanagiya R, Koseki A, Iwamori T, Kudo M (2022). Development of a machine learning-based prediction model for extremely rapid decline in estimated glomerular filtration rate in patients with chronic kidney disease: a retrospective cohort study using a large data set from a hospital in Japan. BMJ Open.

[41] Kaufman HW, Wang C, Wang Y, Han H, Chaudhuri S, Usvyat L (2023). Machine Learning Case Study: Patterns of Kidney Function Decline and Their Association With Clinical Outcomes Within 90 Days After the Initiation of Renal Dialysis. Adv Kidney Dis Health.

[42] Zheng Z, Waikar SS, Schmidt IM, Landis JR, Hsu CY, Shafi T (2021). Subtyping CKD Patients by Consensus Clustering: The Chronic Renal Insufficiency Cohort (CRIC) Study. J Am Soc Nephrol.

[43] Saito H, Yoshimura H, Tanaka K, Kimura H, Watanabe K, Tsubokura M (2024). Predicting CKD progression using time-series clustering and light gradient boosting machines. Sci Rep.

[44] Guo J, Lv Z (2022). Application of Digital Twins in multiple fields. Multimed Tools Appl.

[45] Surian NU, Batagov A, Wu A, Lai WB, Sun Y, Bee YM (2024). A digital twin model incorporating generalized metabolic fluxes to identify and predict chronic kidney disease in type 2 diabetes mellitus. NPJ Digit Med.

[46] Liu W, Wang W, Sun F, Jiang N, Yuan L, Bu X (2024). Machine Learning-Assisted Analysis of Sublingual Microcirculatory Dysfunction for Early Cardiovascular Risk Evaluation and Cardiovascular-Kidney-Metabolic Syndrome Stage in Patients With Type 2 Diabetes Mellitus. Diabetes Metab Res Rev.

[47] Zhu H, Qiao S, Zhao D, Wang K, Wang B, Niu Y (2024). Machine learning model for cardiovascular disease prediction in patients with chronic kidney disease. Front Endocrinol (Lausanne).

[48] Garbelli M, Bellocchio F, Baro Salvador ME, Chermisi M, Rincon Bello A, Godoy IB (2024). The Use of Anemia Control Model Is Associated with Improved Hemoglobin Target Achievement, Lower Rates of Inappropriate Erythropoietin Stimulating Agents, and Severe Anemia among Dialysis Patients. Blood Purif.

[49] Barbieri C, Molina M, Ponce P, Tothova M, Cattinelli I, Ion Titapiccolo J (2016). An international observational study suggests that artificial intelligence for clinical decision support optimizes anemia management in hemodialysis patients. Kidney Int.

[50] Bucalo ML, Barbieri C, Roca S, Ion Titapiccolo J, Ros Romero MS, Ramos R (2018). The anaemia control model: Does it help nephrologists in therapeutic decision-making in the management of anaemia?. Nefrologia (Engl Ed).

[51] Li Z, Liu X, Tang Z, Jin N, Zhang P, Eadon MT (2024). TrajVis: a visual clinical decision support system to translate artificial intelligence trajectory models in the precision management of chronic kidney disease. J Am Med Inform Assoc.

[52] Klamrowski MM, Klein R, McCudden C, Green JR, Rashidi B, White CA (2024). Derivation and Validation of a Machine Learning Model for the Prevention of Unplanned Dialysis. Clin J Am Soc Nephrol.

[53] Chase HS, Radhakrishnan J, Shirazian S, Rao MK, Vawdrey DK (2010). Under-documentation of chronic kidney disease in the electronic health record in outpatients. J Am Med Inform Assoc.

[54] Chan L, Beers K, Yau AA, Chauhan K, Duffy A, Chaudhary K (2020). Natural language processing of electronic health records is superior to billing codes to identify symptom burden in hemodialysis patients. Kidney Int.

[55] Michalopoulos G, Qazi H, Wong A, Butt Z, Chen H (2020). Automatic Extraction of Risk Factors for Dialysis Patients from Clinical Notes Using Natural Language Processing Techniques. Stud Health Technol Inform.

[56] Barr B, Harasemiw O, Gibson IW, Tremblay-Savard O, Tangri N (2023). The Development of a Comprehensive Clinicopathologic Registry for Glomerular Diseases Using Natural Language Processing. Can J Kidney Health Dis.

[57] Singh K, Betensky RA, Wright A, Curhan GC, Bates DW, Waikar SS (2016). A Concept-Wide Association Study of Clinical Notes to Discover New Predictors of Kidney Failure. Clin J Am Soc Nephrol.

[58] Perotte A, Ranganath R, Hirsch JS, Blei D, Elhadad N (2015). Risk prediction for chronic kidney disease progression using heterogeneous electronic health record data and time series analysis. J Am Med Inform Assoc.

[59] Gupta D, Saul M, Gilbertson J (2004). Evaluation of a deidentification (De-Id) software engine to share pathology reports and clinical documents for research. Am J Clin Pathol.

[60] Bajaj S, Gandhi D, Nayar D (2024). Potential Applications and Impact of ChatGPT in Radiology. Acad Radiol.

[61] Underdahl L, Ditri M, Duthely LM (2024). Physician Burnout: Evidence-Based Roadmaps to Prioritizing and Supporting Personal Wellbeing. J Healthc Leadersh.

[62] van Buchem MM, Boosman H, Bauer MP, Kant IMJ, Cammel SA, Steyerberg EW (2021). The digital scribe in clinical practice: a scoping review and research agenda. NPJ Digit Med.

[63] Klann JG, Szolovits P (2009). An intelligent listening framework for capturing encounter notes from a doctor-patient dialog. BMC Med Inform Decis Mak.

[64] Abdelgadir Y, Thongprayoon C, Miao J, Suppadungsuk S, Pham JH, Mao MA (2024). AI integration in nephrology: evaluating ChatGPT for accurate ICD-10 documentation and coding. Front Artif Intell.

[65] Pham JH, Thongprayoon C, Miao J, Suppadungsuk S, Koirala P, Craici IM (2024). Large language model triaging of simulated nephrology patient inbox messages. Front Artif Intell.

[66] Miao JT C, Suppadungsuk S, Garcia Valencia OA, Qureshi F (2023). Innovating Personalized Nephrology Care: Exploring the Potential Utilization of ChatGPT. J Pers Med.

[67] Sarraju A, Bruemmer D, Van Iterson E, Cho L, Rodriguez F, Laffin L (2023). Appropriateness of Cardiovascular Disease Prevention Recommendations Obtained From a Popular Online Chat-Based Artificial Intelligence Model. JAMA.

[68] Haver HL, Ambinder EB, Bahl M, Oluyemi ET, Jeudy J, Yi PH (2023). Appropriateness of Breast Cancer Prevention and Screening Recommendations Provided by ChatGPT. Radiology.

[69] Choi J, Kim JW, Lee YS, Tae JH, Choi SY, Chang IH (2024). Availability of ChatGPT to provide medical information for patients with kidney cancer. Sci Rep.

[70] Qarajeh A, Tangpanithandee S, Thongprayoon C, Suppadungsuk S, Krisanapan P, Aiumtrakul N (2023). AI-Powered Renal Diet Support: Performance of ChatGPT, Bard AI, and Bing Chat. Clin Pract.

[71] Miao J, Thongprayoon C, Suppadungsuk S, Garcia Valencia OA, Cheungpasitporn W (2024). Integrating Retrieval-Augmented Generation with Large Language Models in Nephrology: Advancing Practical Applications. Medicina (Kaunas).

[72] Sheikh MS, Dreesman B, Barreto EF, Thongprayoon C, Miao J, Suppadungsuk S (2024). Identification of kidney-related medications using AI from self-captured pill images. Ren Fail.

[73] Galido PV, Butala S, Chakerian M, Agustines D (2023). A Case Study Demonstrating Applications of ChatGPT in the Clinical Management of Treatment-Resistant Schizophrenia. Cureus.

[74] Ali SR, Dobbs TD, Hutchings HA, Whitaker IS (2023). Using ChatGPT to write patient clinic letters. Lancet Digit Health.

[75] Ayers JW, Poliak A, Dredze M, Leas EC, Zhu Z, Kelley JB (2023). Comparing Physician and Artificial Intelligence Chatbot Responses to Patient Questions Posted to a Public Social Media Forum. JAMA Intern Med.

[76] Garcia Valencia OA, Thongprayoon C, Jadlowiec CC, Mao SA, Leeaphorn N, Budhiraja P (2024). AI-driven translations for kidney transplant equity in Hispanic populations. Sci Rep.

[77] Nayak A, Alkaitis MS, Nayak K, Nikolov M, Weinfurt KP, Schulman K (2023). Comparison of History of Present Illness Summaries Generated by a Chatbot and Senior Internal Medicine Residents. JAMA Intern Med.

[78] Miao J, Thongprayoon C, Cheungpasitporn W (2024). Should Artificial Intelligence Be Used for Physician Documentation to Reduce Burnout?. Kidney360.

[79] Tangri N, Ferguson TW (2022). Artificial Intelligence in the Identification, Management, and Follow-Up of CKD. Kidney360.

[80] Zhao D, Wang W, Tang T, Zhang YY, Yu C (2023). Current progress in artificial intelligence-assisted medical image analysis for chronic kidney disease: A literature review. Comput Struct Biotechnol J.

[81] Acosta JN, Falcone GJ, Rajpurkar P, Topol EJ (2022). Multimodal biomedical AI. Nat Med.

[82] Moor M, Banerjee O, Abad ZSH, Krumholz HM, Leskovec J, Topol EJ (2023). Foundation models for generalist medical artificial intelligence. Nature.

[83] Zhou Y, Chia MA, Wagner SK, Ayhan MS, Williamson DJ, Struyven RR (2023). A foundation model for generalizable disease detection from retinal images. Nature.

[84] Shah K, Xu AY, Sharma Y, Daher M, McDonald C, Diebo BG (2024). Large Language Model Prompting Techniques for Advancement in Clinical Medicine. J Clin Med.

[85] Savage T, Nayak A, Gallo R, Rangan E, Chen JH (2024). Diagnostic reasoning prompts reveal the potential for large language model interpretability in medicine. NPJ Digit Med.

[86] Yang R, Tan TF, Lu W, Thirunavukarasu AJ, Ting DSW, Liu N (2023). Large language models in health care: Development, applications, and challenges. Health Care Sci.

